# Mathematician Yitang Zhang: why did I return to China at 70?

**DOI:** 10.1093/nsr/nwaf370

**Published:** 2025-09-04

**Authors:** Weijie Zhao

**Affiliations:** Weijie Zhao is an NSR news and science editor based in Beijing

## Abstract

*Yitang Zhang (张益唐) emerged as a prominent mathematician in 2013 with his groundbreaking work on the Twin Prime Conjecture, proving that there are infinitely many pairs of primes with gaps less than 70 million. His paper, entitled ‘Bounded Gaps Between Primes’, was accepted by Annals of Mathematics in just 3 weeks—an exceptionally rare feat in the field of mathematics*.

*Overnight, Zhang, a previously obscure 58-year-old lecturer at a US university, became a globally celebrated academic luminary. He was once a top student in Peking University's mathematics department, but after struggling through his PhD program in the USA, he failed to secure an academic position, and worked at a Subway restaurant for 7 years before a friend helped him land a position as an assistant professor at the University of New Hampshire. Until his 2013 breakthrough, he remained a lecturer. This legendary journey earned him the nickname ‘the Hidden Master of Mathematics’*.

*In Chinese martial arts stories, the hidden master often passes on his lifelong skills to a destined young man in his twilight years. When asked by National Science Review* (*NSR*) *why he chose to return to China in 2025 at the age of 70 to work at the Hong Kong Institute of Advanced Study of Sun Yat-sen University, one reason he gave was his hope to pass down the mathematical insights he had accumulated over decades and ‘mentor a group of young talents’ in China*.

*For Zhang, returning to China is about academic legacy, and also a natural choice—he believes China's scientific capabilities are undergoing a qualitative transformation, making it one of the most conducive places for basic research in the world*.

*In this interview with NSR, Zhang also discussed the ancient and captivating field of analytic number theory, his views on mathematical research methods, mathematical education and the temperament of mathematicians*.

## Teaser

An interview of `the Hidden Master of Mathematics', who made breakthrough on the Twin Prime Conjecture in 2013, proving that there are infinitely many pairs of primes with gaps less than 70 million.

## RETURNING TO ROOTS

***NSR*:** You've recently returned full-time to China to work at the Hong Kong Institute of Advanced Study of Sun Yat-sen University (SYSU). Could you share the story behind this decision?

***Zhang*:** I've actually been considering returning for several years. In 2019, at the Future Science Awards ceremony in Beijing, I mentioned that China's rapid advancements in science and technology had me thinking about coming back. After that, several Chinese universities reached out, and I wasn't sure which to choose. Since mathematical research requires minimal conditions, I felt any institution with a solid foundation and being able to provide me with the freedom to focus on my work would suffice.

However, the pandemic delayed my plans. It wasn't until March this year (2025) that SYSU contacted me. Among Chinese universities, they approached me relatively late, but they were very proactive. Shortly after connecting, Professor Zheng'an Yao (姚正安) from the School of Mathematics and several university leaders flew from Guangzhou and Shanghai to Los Angeles to meet with me—their sincerity was evident. I also prefer Guangzhou's warm climate, and my wife was very satisfied with them, so we made the decision relatively quickly. My wife handles much of the external communication for me; our personalities complement each other, which works well.

The Hong Kong Institute of Advanced Study of SYSU is still under construction. For now, I am working at the university's main campus in Guangzhou, but I'll likely be based primarily in Hong Kong later.

**Figure 1:**
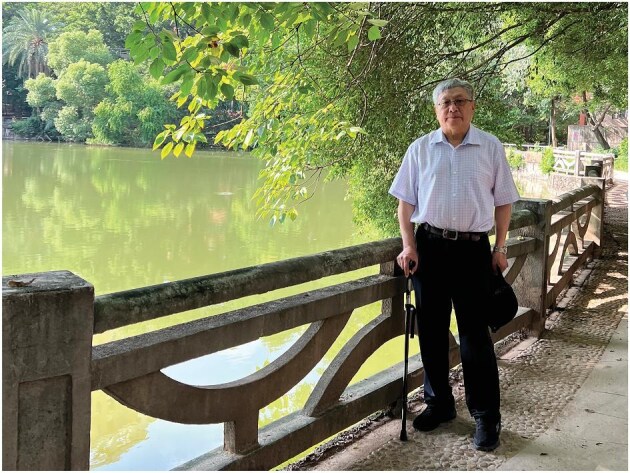
Professor Yitang Zhang at Sun Yat-sen University, Guangzhou, China. (*Courtesy of Prof. Zhang*)

My first visit to SYSU was 41 years ago, in December 1984, when I came from Beijing to attend a conference on number theory. It was winter, freezing in Beijing, but the campus in Guangzhou was still lush and green—it left a lasting impression on me. At the end of that conference, a university leader said to us: ‘Welcome to Sun Yat-sen University!’ Little did I know that 41 years later, those words would come true.

***NSR*:** What were your reasons for considering a return to China? Was it the living environment you preferred?

***Zhang*:** The living environment was certainly a factor, especially since my wife wanted to return and live in China.

But more importantly, over the past decade since my breakthrough, I've often reflected: if I spent the rest of my life at a foreign university, it would be OK, but somewhat meaningless. Meanwhile, China's scientific progress has been rapid, with many outstanding young talents emerging. Our foundational education in primary and secondary schools, while still having room for improvement, is also more robust than in the USA, allowing gifted children to stand out. I believe that in theoretical sciences, China will undoubtedly rise to the top of the world.

Given this momentum, I wanted to return and to mentor a group of young talents. I hope to guide China's brightest young minds and pass on the insights I've accumulated over decades.

I hope to guide China's brightest young minds and pass on the insights I've accumulated over decades.

—Yitang Zhang

***NSR*:** What are your research and mentoring plans in SYSU?

***Zhang*:** I currently have an important paper in progress on Landau–Siegel zeros, which is nearly complete. I hope to finalize it at SYSU—it shouldn't take long.

For graduate training, I aim to find students genuinely interested in analytic number theory, with solid foundational skills and a willingness to delve deep. I want to engage with them extensively, sharing my mathematical perspectives. Whether they're from SYSU or elsewhere, I welcome them all.

When mentoring graduate students, I always treat them as friends, discussing problems openly. Young people often have fresh, dynamic ideas, which will not be uncovered unless you frequently communicate with them. I hope to spark intellectual exchanges with them, without rigid formalities.

***NSR*:** What if a student you admit later fails to meet your expectations?

***Zhang*:** If a student's interests shift toward another direction, I would respect his or her choice. Being the mentor, I would still try to help them with job searches or other aspects.

***NSR*:** Will you teach undergraduate courses?

***Zhang*:** I likely will not lecture the large undergraduate classes, but will lead graduate seminars. At the graduate level, mathematics courses are primarily discussion-based. Sometimes I'll present; other times, I'll assign a paper for students to analyze and present.

## NUMBER THEORY: THE OLDEST, HARDEST AND MOST VITAL BRANCH OF MATHEMATICS

***NSR*:** What is number theory?

***Zhang*:** Number theory studies integers—it's arguably the oldest, most challenging and most enduring branch of mathematics. My research focuses on the famous unsolved conjectures about integer properties, particularly prime properties, such as the Goldbach Conjecture and the Twin Prime Conjecture. These belong to the classical side of number theory, tackling problems posed centuries or even millennia ago that remain unresolved due to their difficulty.

Number theory also includes newer areas like the Langlands Program and arithmetic geometry, which employ modern tools but often circle back to classical problems.

Professor Shou-Wu Zhang of Princeton once divided number theory into two parts: classical number theory, which focuses on primes; and the study of solutions to polynomial equations or systems of equations—to see whether the equations have integer solutions or rational solutions. These two parts diverge but also intersect in many ways—for instance, equation research often ties to algebraic geometry, which in turn applies to prime distribution.

***NSR*:** What is analytic number theory?

***Zhang*:** Analytic number theory uses methods from function theory, particularly complex analysis, to study number theory. Problems like the Goldbach Conjecture and Twin Prime Conjecture currently seem approachable only through analytic methods, which is why my work falls under the umbrella of analytic number theory.

***NSR*:** Please give several examples of the major conjectures in analytic number theory.

***Zhang*:** Firstly there is the Riemann Hypothesis, which is the most important and most challenging problem in not only number theory, but also all of mathematics. It concerns the Riemann zeta function, a complex function that is taught in undergraduate courses. The hypothesis states that all zeros of this function lie on the vertical line in the complex plane where the real part equals 1/2. That is to say, the function can only equal zero when the real part is exactly 1/2. What is particularly interesting is that the Riemann Hypothesis also relates to the study of prime numbers, and the two can mutually reinforce each other.

The Landau–Siegel Zero Conjecture, which I am working on, is a special case of the Riemann Hypothesis. I aim to prove the Landau–Siegel zeros don't exist, which means that a certain class of functions never equal zero at a specific point.

Other famous conjectures about primes include the Goldbach Conjecture, the Twin Prime Conjecture and many others. For example, a conjecture asserts there are infinitely many primes that can be written in the form of a^2^ + 1, e.g. 4^2^ + 1 = 17, and 17 is a prime, 6^2^ + 1 = 37, and 37 is a prime, and so on. The conjecture states that there are infinitely many such primes.

Primes are simple to define, but conjectures about their properties are notoriously hard—most remain unsolved and are awaiting breakthroughs.

***NSR*:** How does number theory connect with other mathematical fields?

***Zhang*:** Methodologically and conceptually, number theory intertwines strongly with other disciplines. Results from fields like algebraic geometry can unexpectedly advance number theory—which has happened more than once in the history. These cross-disciplinary links are fascinating.

***NSR*:** Some say mathematics is ‘numbers, shapes, plus logic’. Do you agree?

***Zhang*:** I agree—this statement is always right. It provides a definition for mathematics in a simple way. But it's also overly simple and broad, lacking specifics.

Primes are simple to define, but conjectures about their properties are notoriously hard —most remain unsolved and are awaiting breakthroughs.

—Yitang Zhang

***NSR*:** Do you have academic idols?

***Zhang*:** In analytic number theory, I admire three: Soviet mathematician Ivan Matveyevich Vinogradov (1891–1983), Norwegian mathematician Atle Selberg (1917–2007) and China's Jingrun Chen (陈景润, 1933–1996).

The mathematics of the former Soviet Union was once something the West feared. Back then, there was very little academic exchange between the Soviet Union and the West, but from time to time, a mathematical genius would emerge from the Soviet Union and turn the mathematical world upside down.

The field of analytic number theory was largely developed around the 1920s by British mathematicians such as Godfrey Harold Hardy (1877–1947) and John Edensor Littlewood (1885–1977). The mathematicians who laid the foundation for analytic number theory in China, including Luogeng Hua (华罗庚), Sihe Min (闵嗣鹤) and Zhao Ke (柯召), were all trained in the UK.

But in the 1930s, out of nowhere, the Soviet Union produced Vinogradov. Using trigonometric sum estimates, also known as exponential sum estimates, he solved the Three Primes Theorem and suddenly pushed Soviet analytic number theory to its peak, even surpassing the UK. He introduced and developed a whole new set of methods. Looking back now, these methods might seem relatively simple, but they truly made immense contributions to and greatly advanced number theory.

The second person I admire is Norway's Selberg. Initially, in the 1940s when Norway had not yet been occupied by Nazi Germany, he conducted analytic number theory research entirely on his own and independently. After World War II ended, a series of his results were published, one after another. He made breakthrough works in sieve methods and other areas, improving the earlier work of Hardy and Littlewood. Selberg was awarded the Fields Medal in 1950.

The third is Jingrun Chen. Chen's contributions actually aren't limited to proving the ‘1 + 2’ of the Goldbach Conjecture; he had pioneering ideas and worked in many other areas as well. Some of his work still hasn't been fully understood to this day and has room for further development. The reviewer of my twin prime paper, Professor Henryk Iwaniec of Polish origin, also greatly admires Chen.

Moreover, under the harsh living conditions at the time, Chen's perseverance in persistently pursuing mathematical research is something we can't help but admire. I still remember when I had just been admitted to Peking University, I heard about Chen's work and really wanted to read his original paper. A colleague of my father helped me borrow a copy of the journal *Scientia Sinica* (in Chinese), which contained Chen's paper entitled ‘On the representation of a large even integer as the sum of a prime and the product of at most two primes’—I still remember the title to this day. At the time, I certainly couldn't fully understand it, but I could grasp some parts and generally understood the work. Seeing that a Chinese could produce such great mathematical achievement, I was very excited and thought to myself: could I also produce results as good as this in the future?

Mentioning Chen, it reminds me of another question: are Chinese people particularly good at calculation? Chen was especially skilled at calculation. In Peking University, my teachers also said I was particularly good at calculation. On mathematical problems, we aren't satisfied with conceptual understanding; we always want to calculate it concretely and see which number is the solution. This might be something I am comparatively good at, and it's also one reason I was able to make a breakthrough on the Twin Prime Conjecture. If Chinese people really have this kind of innate strength, perhaps we can continue to leverage it in the future to achieve more results.

**Figure 2:**
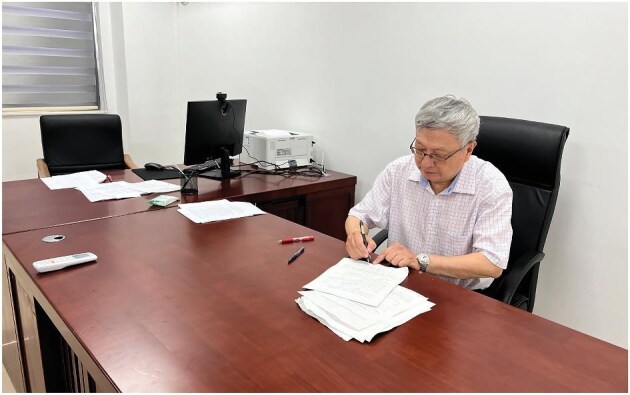
Mathematical research requires minimal conditions—Professor Yitang Zhang working in his new office in SYSU, Guangzhou. *(Courtesy of Prof. Zhang)*

***NSR*:** If Chinese are good at calculation, what are other countries particularly skilled at?

***Zhang*:** For example, the French school of the last century didn't focus much on concrete calculation but preferred developing concepts. The set of concepts they established was extremely powerful. The mathematical aptitudes of different countries—it's not about who is superior or inferior, but the ways they manifest might differ somewhat.

As mentioned earlier, China's analytic number theory school originally inherited from Britain, and the British school was also skilled at calculation. The 2022 Fields Medalist James Maynard [born in 1987; in 2014 he proved there are infinitely many pairs of primes differing by at most 246, advancing Zhang's result] is also British and is very strong in this aspect.

## MATHEMATICAL RESEARCH: FROM PEN AND PAPER TO ARTIFICIAL INTELLIGENCE

***NSR*:** How do you perform your research? Do you only need pen and paper? How can computers help?

***Zhang*:** For me, computers are primarily an auxiliary tool. I need e-mail to communicate with others, and I also use computers to write and print papers. Decades ago, when Jingrun Chen conducted his research, everything had to be handwritten, which was extremely arduous. It is said that his draft papers filled several sacks. When relying solely on handwriting, if any part needed revision, the entire document had to be rewritten. Now, with computers, such revisions are much easier.

Additionally, I also use several forms of calculation software. Some calculations, such as matrix computations, are quite difficult to do solely by hand, but are very straightforward for computers.

***NSR*:** Do other branches of mathematics rely more on computational tools?

***Zhang*:** In fields like applied mathematics, the main work involves modeling and computation. For example, some of my colleagues work on building earthquake models, and such extensive computational tasks are primarily carried out by computers.

If we are talking about computation itself being used to help solve a major theoretical mathematical problem, one of the most iconic examples is the sensational solution of the Four Color Problem in 1976. The Four Color Problem sought to prove that for any map, only four colors are needed to distinguish adjacent regions. In 1976, two mathematicians from the University of Illinois Urbana-Champaign, Kenneth Appel and Wolfgang Haken, categorized this problem into tens of billions of different cases. They applied for and received support from their university, using its high-performance computer to perform 1200 h of calculations, proving each case individually and ultimately solving the Four Color Problem.

… are Chinese people particularly good at calculation? … we aren't satisfied with conceptual understanding; we always want to calculate it concretely and see which number is the solution.

—Yitang Zhang

This was a landmark event, demonstrating that computers could replace human effort in completing parts of mathematical proofs. However, to this day, computers still cannot replace humans in conducting all mathematical proofs.

***NSR*:** Recently, there have also been rumors that an artificial intelligence (AI) company in Shenzhen has completely resolved the Twin Prime Conjecture. Have you heard about this? What are your views on the application of AI in the field of mathematics?

***Zhang***: I have not yet seen any related article, so I cannot comment casually. However, personally, I am currently somewhat skeptical that AI can fully crack problems like the Twin Prime Conjecture. Of course, I could be wrong. AI will undoubtedly see more developments in the future, but I cannot predict the specifics.

***NSR*:** Is the underlying principle of AI a mathematical problem?

***Zhang*:** I suppose so, because it is ultimately a matter of logic.

## MATHEMATICS EDUCATION: HOW TO BUILD A SOLID FOUNDATION WHILE REDUCING THE BURDEN

***NSR*:** Regarding the cultivation of China's next generation mathematicians, do you have any specific suggestions?

***Zhang*:** I think firstly, as the ancient Chinese saying goes: Do not unjustly belittle yourself. In terms of soft power or scientific research, the strength comparison between China and the USA is changing. As long as we focus on what we are good at and devote ourselves to our work, there will surely be continuous new achievements.

However, on the other hand, we must also emphasize the importance of working solidly and avoid impetuosity. Do not exaggerate your work, claiming too readily that we have produced some earth-shattering, revolutionary result. I believe our younger generation can indeed produce excellent work, but such work must be earned through effort, not empty boasts. Professor Shing-Tung Yao (丘成桐) and many other mathematicians have expressed similar views: we must avoid impetuosity.

In recent years, China has cultivated some outstanding mathematical talent, but we need not always be in a rush to wonder whether they can win some major mathematical prize. Winning awards is, of course, a good thing and would boost the development of mathematics in China. However, awards should be the natural outcome that follows the overall improvement of our strength.

***NSR*:** Will Hong Wang (王虹) win the Fields Medal? [Hong Wang was born in 1991, awarded Bachelor of Mathematics from Peking University, and PhD from MIT. She is currently Associate Professor at New York University; in 2025, together with Joshua Zahl, she resolved the Kakeya Conjecture in three-dimensional space.]

***Zhang*:** Very likely. I sincerely hope she will win the award in 2026, and given her young age, even if she does not win it this time, she will still have another opportunity 4 years later.

***NSR*:** Many other research disciplines often say they need more young people to join their fields. Is mathematics different in that it does not require many people?

***Zhang*:** Yes, mathematics does not require many people. Some have suggested that if all the top high-school teachers from one province in China were gathered together, they can solve the ‘1 + 2’ problem. No, they cannot. Mathematical problems cannot be solved by solely increasing the number of researchers. Expensive experimental equipment or large-scale laboratories are also not required.

As my advisor in Peking University, Chengbiao Pan (潘承彪), said many years ago, mathematical research is often about finding joy in one's own work. It is meticulous work that requires one to bury oneself in it.

I hope that mathematical research does not become too ‘noisy’; that is not what mathematics should be like.

Mathematical problems cannot be solved by solely increasing the number of researchers.

—Yitang Zhang

***NSR*:** Is China's current education system suitable for the growth of mathematicians?

***Zhang*:** I think it is essentially suitable. China's fundamental education is relatively intensive, and coupled with China's large population, it is able to identify intelligent children with mathematical talent. However, on the other hand, such an education also has side effects: for ordinary primary and secondary school students, it may create an excessive burden. As for how to balance these two aspects, I do not have a good solution.

Winning awards in competitions does not necessarily mean one can become an outstanding mathematician. The Soviet Union was a major power in mathematics and won many International Mathematical Olympiad (IMO) gold medals. The famous Soviet mathematician Andrey Kolmogorov also recognized this problem and personally mentored those award-winning children, guiding them toward high-level research, and

China's fundamental education is relatively intensive, and coupled with China's large population, it is able to identify intelligent children with mathematical talent.

—Yitang Zhang

ultimately, he did cultivate some outstanding mathematicians. I think these experiences are something we can learn from.

***NSR*:** How can we cultivate the IMO champions, who are good at taking exams, into true mathematicians?

***Zhang*:** This is indeed a question that all of us mathematicians should consider. I believe a relatively important point is that if you are already an established mathematician, it is best not to just do your own research, but also practically mentor the young people and help them grow.

In school education and during competitions, we require children to provide the ‘correct answers’ and avoid mistakes. However, in real mathematical research, no one can judge whether a conjecture or hypothesis is correct or wrong at the beginning. Only by truly attempting and delving into it can breakthroughs be made. Therefore, we must help children break through these limitations and not be confined by the ‘correct answers’, daring to explore freely.

***NSR*:** For researchers in other disciplines, do you think the mathematics education they received is sufficient?

***Zhang*:** I think the mathematical literacy of researchers in other disciplines should actually be further strengthened.

I once received an e-mail from a Silicon Valley IT engineer. He said he had studied a formula in number theory and used a computer to verify that the formula is correct for tens of billions of numbers. He asked whether this could prove the formula was correct and whether it could be considered a new discovery.

I was very surprised, because for the formula he mentioned, a very simple mathematical induction method—which high-school students can master—could prove it holds for all integers. There was no need to verify tens of billions of numbers.

Therefore, I believe that if IT practitioners and researchers in many other disciplines had a more solid foundation in mathematics, they would likely be more adept in their respective professional fields.

China's high-school mathematics education has always had high requirements, but a few years ago, there were some changes, and they even tried to remove mathematical induction from high-school textbooks. When I heard this news, I was very surprised, and I firmly oppose this approach. Professor Boju Jiang (姜伯驹) from Peking University and others also voiced opposition, arguing that primary and secondary-school mathematics education relates to the basic quality of the nation's people and even to the rejuvenation of the Chinese nation. I agree with these views and hope that China's basic mathematics education can continue to be strengthened, instead of weakened.

## ARE MATHEMATICIANS BORN?

***NSR*:** From our previous conversation, it seems that you are intimately familiar with the stories of many mathematicians. Have you specifically studied the history of mathematics?

***Zhang*:** I have a great interest in the history of mathematics, but I have not conducted research on it, and I am certainly no expert. The same goes for my other hobbies—I am merely an amateur enthusiast.

***NSR*:** You have interests in music, literature and other fields. When did you develop these hobbies?

***Zhang*:** There aren't too many things that I like, but once I develop an interest in something, I become deeply absorbed in it. This is true for mathematics, and it's also true for my other hobbies.

My interest in literature began early. When I was in my teens, I came across the complete collections of Tang and Song poetry, which were published after the liberation of China, and I was immediately captivated. My fondness for Li Bai and Du Fu started from that time.

My interest in music probably came relatively later. But actually, I greatly enjoyed some of the then-popular revolutionary songs when I was a teenager—I found them very beautiful, and even now I still remember and like some of them. My exposure to classical music came later. When I first heard Beethoven's Sixth Symphony, the Pastoral Symphony, I was utterly enchanted. I felt this is music created by God; humans could not have made such music.

***NSR*:** Is there any common ground between mathematics and these hobbies?

***Zhang*:** Others have asked me this question before. If there is a common characteristic, it is that they are all beautiful and worthy of one's affection. However, if someone suggests that listening to music helped me excel in mathematics, or that excelling in mathematics allowed me to better appreciate music—there doesn't seem to be such a direct connection, as they are, after all, different fields.

Some of my friends have said that my defining characteristic is that my heart is exceptionally calm.

—Yitang Zhang

***NSR*:** Is it that if a person possesses certain inherent traits, such as being able to enjoy quiet and contemplation, he or she would be more likely to develop an affinity for these things?

***Zhang*:** Yes, I agree with this point. If a person is not impetuous, if his or her heart can remain calm, he or she would be more likely to appreciate these things and more likely to delve deeply into mathematical problems. Some of my friends have said that my defining characteristic is that my heart is exceptionally calm. Older generations of Chinese mathematicians, such as Luogeng Hua, also possessed considerable cultivation in areas like classical literature.

***NSR*:** Are these traits innate? Are mathematicians born?

***Zhang*:** For myself, it might indeed be innate. I have been this way since childhood; it wasn't something others specifically cultivated in me.

***NSR*:** One last question: do you have any advice for young students?

***Zhang*:** I don't think I am qualified to offer advice to young students. But based on the path I have walked through, I hope young people can truly have lofty aspirations. If you genuinely like something, do not give up on it easily. Life is finite, if you truly wish to achieve something of great value—whether in academia or other fields—you should persevere in that direction.

